# A Polymeric Piezoelectric Tactile Sensor Fabricated by 3D Printing and Laser Micromachining for Hardness Differentiation during Palpation

**DOI:** 10.3390/mi13122164

**Published:** 2022-12-07

**Authors:** Chang Ge, Edmond Cretu

**Affiliations:** Department of Electrical and Computer Engineering, The University of British Columbia, Vancouver, BC V6T 1Z4, Canada

**Keywords:** piezoelectric tactile sensor, palpation, laser micromachining, elastomer 3D printing, bionic MEMS, tissue hardness differentiation

## Abstract

Tactile sensors are important bionic microelectromechanical systems that are used to implement an artificial sense of touch for medical electronics. Compared with the natural sense of touch, this artificial sense of touch provides more quantitative information, augmenting the objective aspects of several medical operations, such as palpation-based diagnosis. Tactile sensors can be effectively used for hardness differentiation during the palpation process. Since palpation requires direct physical contact with patients, medical safety concerns are alleviated if the sensors used can be made disposable. In this respect, the low-cost, rapid fabrication of tactile sensors based on polymers is a possible alternative. The present work uses the 3D printing of elastic resins and the laser micromachining of piezoelectric polymeric films to make a low-cost tactile sensor for hardness differentiation through palpation. The fabricated tactile sensor has a sensitivity of 1.52 V/mm to mechanical deformation at the vertical direction, a sensitivity of 11.72 mV/HA in sensing material hardness with a pressing depth of 500 µm for palpation, and a validated capability to detect rigid objects buried in a soft tissue phantom. Its performance is comparable with existing piezoelectric tactile sensors for similar applications. In addition, the tactile sensor has the additional advantage of providing a simpler microfabrication process.

## 1. Introduction

Palpation is a simple medical technique with high effectiveness, vast applications, and a long history [1]. In ancient times, medical professionals used palpation as a convenient tool for diagnosis. Nowadays, palpation methods are still used, combined with other medical equipment, to enhance the feedback provided to the surgeon in procedures such as open surgeries [1,2,3,4]. Palpation detects local physical differences in the hardness of tissue layers. Medical professionals associate such subjective information with specific diagnostic procedures. The quality of a classic palpation technique heavily depends on a human’s natural sense of touch, which is a subjective measure with high inter-variability from person to person. It is challenging to conduct accurate tissue hardness differentiation with a high resolution using only human hands. It may take medical professionals years or decades to master the required skills. As revealed in the existing research [5,6], one effective way to tackle this challenge is using medical systems with tactile sensors to conduct palpation operations.

Tactile sensors are an important category of bionic microelectromechanical systems (MEMS). They are the basis for the artificial sense of touch [7]. Compared with its natural counterpart based on human hands, the artificial sense of touch based on tactile sensors can obtain more quantitative information at a higher resolution, with the potential of recording objective data that can be later interfaced with an artificial intelligence layer for augmented medical diagnostic procedures. Electronic systems with tactile sensors can significantly reduce the challenge of the accurate hardness of differentiation during palpation. Motivated by this unique advantage, various types of tactile sensors have been developed to support palpation techniques [8,9,10,11,12]. The application potential of these MEMS devices, especially in minimally invasive surgery and catheter-based diagnosis, has been extensively demonstrated. One current research focus on this topic is the development of disposable tactile sensors to address concerns related to medical safety. Such systems will have a reusable electronics module and a disposable front end consisting of tactile sensors that are in touch with the patient during the palpation procedure. Such an approach can significantly minimize the risk of patient-to-patient contaminations during the sequential use of robot-assisted palpations.

The need for disposable tactile sensors for palpation has introduced two demands on microfabrication technology. Firstly, the manufacturing process of tactile sensors should be simple and, therefore, robust and low-cost. Secondly, the fabrication technology should produce tactile sensors with acceptable performances for palpation techniques. The authors have previously developed a rapid manufacturing technology for polymeric piezoelectric MEMS transducers [13]. The technology uses 3D printing to fabricate mechanical structures and laser micromachining to pattern piezoelectric polyvinylidene fluoride co-trifluoroethylene (PVDF-TrFE) thin films. By replacing the traditional layer-by-layer micromachining sequential strategy with these one-step material processing methods, the overall complexity in the manufacturing of piezoelectric MEMS transducers is significantly reduced. The robustness of this manufacturing technology has previously been validated by making piezoelectric resonating MEMS mass sensors [13]. This paper examines the possibility of using this rapid prototyping technology to develop piezoelectric tactile sensors for palpation-based tissue hardness differentiation. A piezoelectric tactile sensor was designed and fabricated using the rapid prototyping technology developed in the authors’ previous research [13]. The fabricated sensor has been tested for its performance in palpation-based tissue hardness differentiation. The corresponding result shows that the performance of the palpation sensor is comparable with other piezoelectric tactile sensors, indicating the promising application potential of the authors’ rapid prototyping technology and the manufactured piezoelectric tactile sensor in low-cost or disposable intelligent medical tools.

For the rest of the paper, Section 2 provides details on the design and fabrication of the piezoelectric tactile sensor for tissue hardness identification during palpation. Section 3 presents the characterization and test procedures used for the piezoelectric tactile sensors. Section 4 shows the characterization results and their interpretation. Section 5 summarizes the paper. The scope of future work is also included.

## 2. Design and Fabrication of the Tactile Sensor

### 2.1. Structure Design

**Figure 1:**
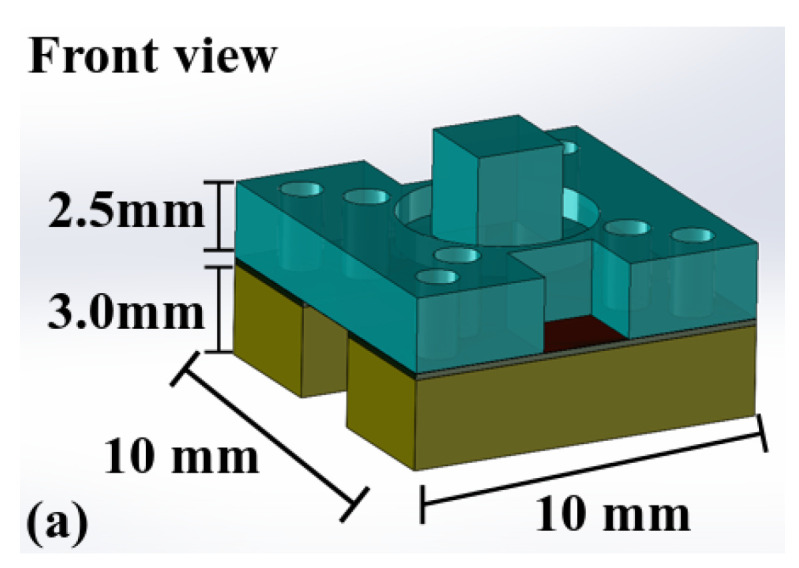

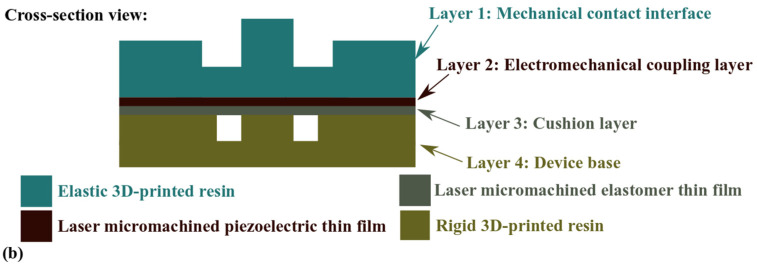
The general structure of the polymeric piezoelectric tactile sensor. (**a**) Front view, (**b**) Cross-section view.

The front and cross-section views of the tactile sensor are shown in Figure 1.

Figure 1a shows the overall dimensions of the tactile sensor. The tactile sensor designed here has a planar dimension of 10 mm by 10 mm (with an active sensing area, in this case, 3 mm by 3 mm). In comparison, the diameter of some endoscopes for minimally invasive surgery is typically in the range of 10.8–13 mm [5,6]. Another reported tactile sensor, using the shift of peaks on the electrical impedance-frequency spectrum to differentiate between the levels of tissue hardness, has a similar size [9]. Hence, for the proof-of-concept aimed in this work, the dimensional design of the tactile sensor can be considered appropriate. The cross-section view in Figure 1b provides detail on the structural configuration of the tactile sensor. The tactile sensor has four polymeric layers. Layer 1 and Layer 4 are made by 3D printing techniques, while Layer 2 and Layer 3 are fabricated by laser micromachining. By using these two direct processing techniques, the fabrication process of the tactile sensor gains extra simplicity when compared with the more traditional micromachining methods.

Most micromachining techniques for fabricating MEMS devices rely on the iteration of a 3-step core cycle of (1) Material deposition, (2) Masking lithography based on photosensitive polymers, and (3) Anisotropic/selective etching. The fabrication flow of MEMS devices can be simplified if one or more steps in this core cycle are skipped during the manufacturing process of microstructures. It is extremely challenging to reach this goal for MEMS fabrication based on silicon or metallic materials. However, the situation becomes different for polymer-based MEMS structures due to some intrinsic material and physicochemical properties.

Because some polymers are photosensitive, photolithographic techniques can directly structure device layers without supplementary masking and sacrificial layers. Two representative technologies in this respect are stereolithography (for 3D printing) [14] and grayscale lithography [15,16,17]. Both techniques can form polymeric structures with multiple degrees of freedom without using sacrificial layers and their associated micromachining steps. The involvement of extra material deposition and the anisotropic/selective etching processes are minimized or eliminated, increasing the simplicity of the fabrication flow for polymeric MEMS devices. These advantages are the basis for using 3D printing processes to fabricate Layer 1 and Layer 4 in Figure 1b. Though Layer 1 in Figure 1b can also be manufactured by molding or hot embossing the processes of elastomers, such as polydimethylsiloxane (PDMS), the preparation process of the molds in these two methods would require additional steps. The direct 3D printing of the elastomer layers reduces the process complexity.

Secondly, the decomposition temperature of the polymers is usually significantly lower than that of silicon or metallic materials. Therefore, it becomes possible to use some serial processing methods to directly shape the microstructures into thin polymer films at an acceptable speed. Two representative examples of such manufacturing techniques are electrical discharge micromachining (EDM) [18] and laser micromachining [19]. When these two processes are used to make polymer MEMS devices from polymer thin films, there are no extra material deposition and masking lithography steps, further reducing the process’s complexity. These advantages are the primary motivation for using the laser micromachining technique to fabricate the electromechanical coupling layer and the cushion layer in Figure 1b.

**Figure 2:**
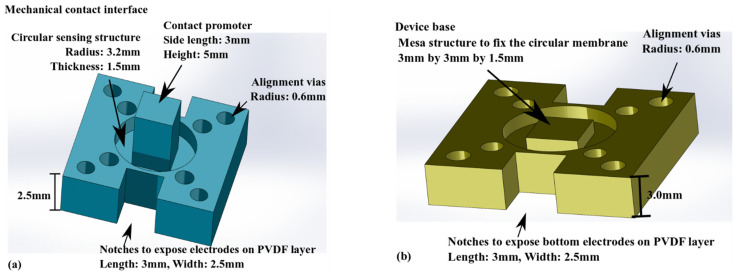
Detailed designs of structures to be 3D printed. (**a**) The elastic contact interface, (**b**) The device base.

**Figure 3:**
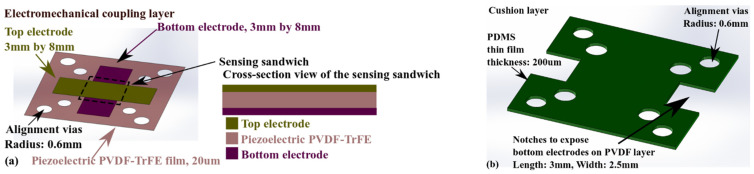
Detailed designs of structures for laser micromachining. (**a**) The piezoelectric PVDF-TrFE layer, (**b**) The PDMS cushion layer.

Figure 2 and Figure 3 present the detailed design of the four layers in Figure 1b.

The two designs in Figure 2 for 3D printing have two common characteristics. Alignment vias are used to accurately position each layer on top of the others during the assembly process, to form the device shown in Figure 1. The notches help to expose the electrodes on the PVDF thin films for external interconnects. As for the differences in the two designs, the 3D printed top elastic layer in Figure 2a features a 3 mm-by-3 mm-by-5 mm contact promoter (as the active palpation area) at its center. Around the promoter is a circular region with a radius of 3.2 mm and a thickness of 1.5 mm. The rest of the top elastic layer has a 2.5 mm thickness to minimize the risk of curling during 3D printing. Figure 2b shows a 3 mm-by-3 mm square mesa with a thickness of 1.5 mm at the center of the base. This square mesa provides a protective fixed constraint. Palpation procedures can lead to large deformations, reaching a few hundred micrometers [10,20]. The mesa in the device base layer has the role of minimizing the risk of structural damage.

Similar to the structural parts in Figure 2, the two components in Figure 3 have corresponding alignment vias. As shown in Figure 3a, the mechano-electrical coupling of the tactile sensor is achieved by using a 20 µm-thick piezoelectric PVDF-TrFE film. This material has higher thermal stability than pure PVDF polymer [21]. Two electrode layers deposited (by screen printing) on the bottom and top surfaces of the piezoelectric film overlap the center area to create a 3 mm-by-3 mm sensing structure. The PDMS cushion layer in Figure 3b separates the PVDF-TrFE layer from the device base, minimizing the risk of cracking the piezoelectric layer during an operation. 

### 2.2. Device Fabrication

**Figure 4:**
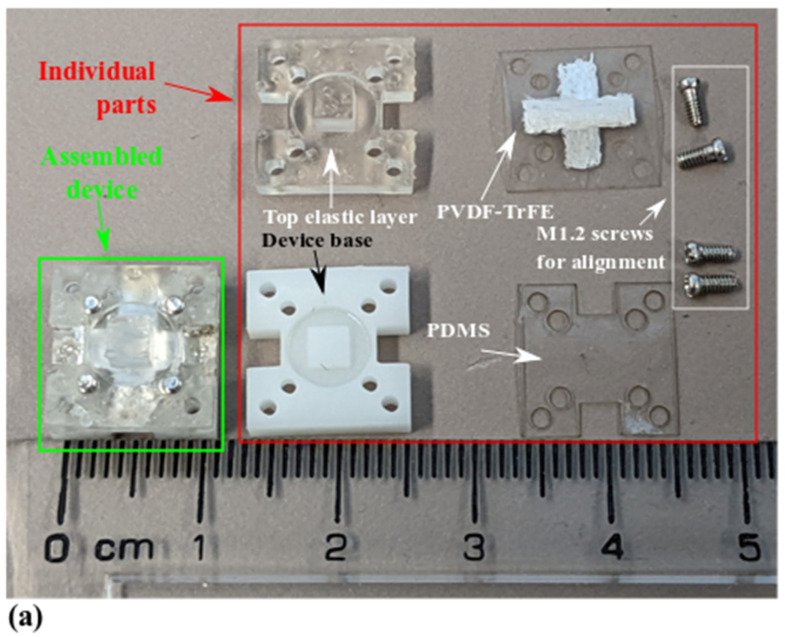

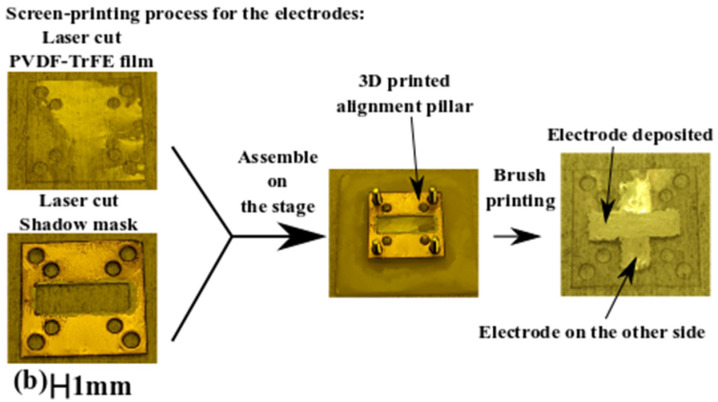
Fabricated device and parts. (**a**) An assembled tactile sensor and separate individual components; (**b**) Screen printing process to make the electrode layers on the bottom and top surface of the PVDF-TrFE film.

The design introduced in Section 2.1 was fabricated using a rapid prototyping technology previously developed by the authors [13]. Table 1 summarizes the manufacturing information for the key components. The fabricated device and components are shown in Figure 4.

In Figure 4a, the individual parts (in the red frame) are assembled into the piezoelectric tactile sensor (in the green frame) based on a sequence of steps. Firstly, the PVDF-TrFE layer is combined with the PDMS layer through roll-to-roll adhesive lamination, using 25 wt% polypropylene carbonate (PPC)/acetone solution as an adhesive. Then, the screws in Figure 4a are partially pushed into the device base from the bottom side to form alignment pillars. After applying the PPC solution onto the device base and the bottom surface of the top elastic layer, the PVDF/PDMS composite is aligned with the four screws and placed on the device base. The top elastic layer is placed onto the device base afterward. As the last step, the screws are fully pushed into the device base. Figure 4b shows the details of how shadow masks are used to create electrodes during the screen-printing process. This electrode fabrication process differs from the authors’ previous work [13], where the E-beam evaporation of thin aluminum films was used. It shows that the manufacturing process previously developed by the authors has a level of flexibility by allowing alternative techniques for some of the fabrication steps. 

## 3. Characterizations of the 3D Printed Tactile Sensor

### 3.1. Experiment Setup

**Figure 5:**
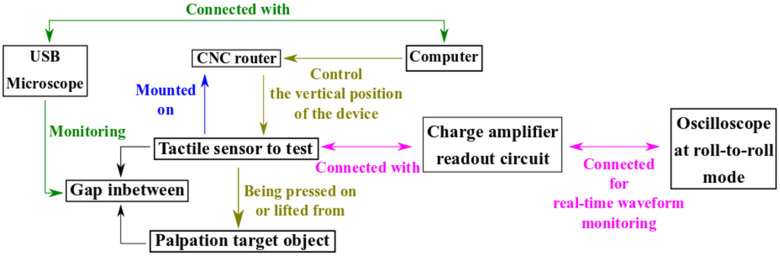
Schematic of the hardware setup to test the fabricated tactile sensor.

Figure 5 illustrates the generic setup used to test the piezoelectric tactile sensor for hardness differentiation during palpation. The equipment is listed in Table 2.

As shown in Figure 5, the palpation operation consists of repeated cycles that involve lowering the *Z*-axis of the CNC router to press the sensor onto an object before raising it to release the sensor from the object. The USB microscope ensures that each palpation process is conducted at a proper initial position. Here, a proper initial position is identified as the *Z*-axis coordinate, where the gap between the tactile sensor and the target object is almost invisible in the image of the USB microscope. The reported *Z*-axis positioning resolution of the Genmistu^®^3018-Pro CNC router (in Table 2) is 0.001 mm. The charge readout circuit in Figure 5 uses two Analog Device^®^ AD8606 amplifiers on a CN0350 piezoelectric sensor evaluation board. According to the user manual of CN0350, the readout voltage can be expressed as:

(1) $$V_{out}=V_{REF}+\frac{Q_{piezo}}{C_{2}}$$

In Equation (1), *V_out_* is the output of the second AD8606 amplifier. *Q_piezo_* is the polarized charge generated during tactile sensing. C_2_ has a value of 1 nF. The DC power supply (listed in Table 2) provides a 3.3 V DC voltage for the readout evaluation board. According to the user manual of CN0350, the corresponding output voltage has a minimum value of around 40–60 mV. The output voltage range is from 0 V to 2.5 V. The DC offset of the output, *V_REF_*, is around 1.5 V. Three types of tests have been conducted using the setup shown in Figure 5 and Table 2. Their setup is introduced in detail in the following sections.

### 3.2. Sensitivity to Pressing Depth during Palpation

**Figure 6:**
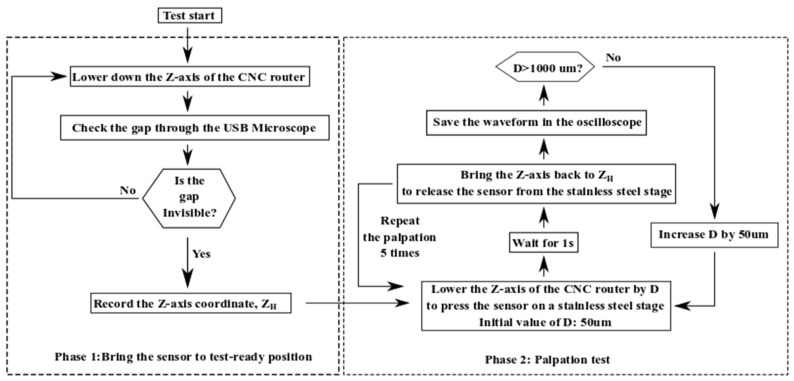
The procedure to measure the tactile sensor’s sensitivity to pressing depth during palpation.

The pressing depth during palpation is one of the two primary factors affecting the output of a tactile sensor. For the sensor fabricated in Section 2, its sensitivity to the pressing depth has been tested using the procedure illustrated in Figure 6.

As shown in Figure 6, the palpations are conducted at a fixed location on a stainless-steel stage. The initial vertical position of the sensor is optimized only once during Phase 1. The vertical position to initiate palpations, Z_H_, is kept the same throughout the characterization. During Phase 2 in Figure 6, the average value of the voltage outputs of five successive measurement cycles is considered as the voltage readout corresponding to a given *Z*-axis displacement D. The measurement process increases D from 50 µm to 1000 µm. The corresponding increment step is 50 µm. The voltage readout is the measure of the charges induced by the direct piezoelectric effect of the PVDF-TrFE film. Since the stainless-steel stage has a significantly higher stiffness than the tactile sensor’s elastic structures, the deformation of the stainless-steel stage during the palpations is neglected. In addition to evaluating the tactile sensor’s sensitivity to the pressing depth during palpation, another purpose for using such a wide testing range in Figure 6 is to determine a proper pressing depth for the other two tests. Here, a proper pressing depth is defined as the *Z*-axis displacement that will not lead to irreversible deformation and structural damage to the tactile sensor.

### 3.3. Sensitivity to Material Hardness

**Figure 7:**
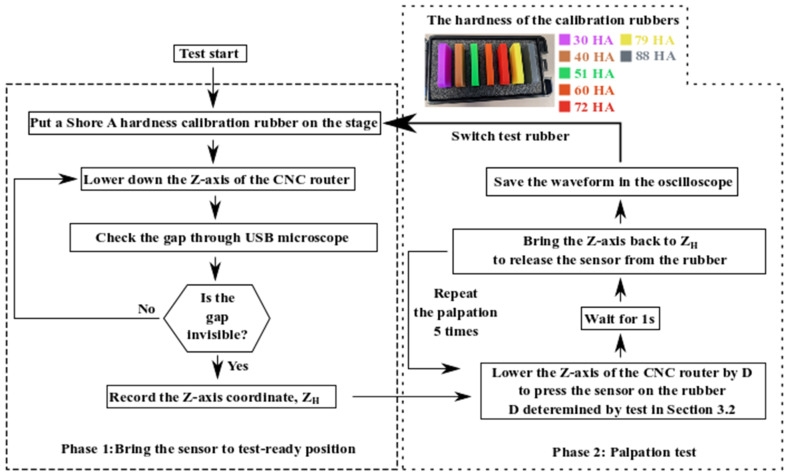
The method to test the tactile sensor sensitivity to material hardness.

The hardness of the target object is the other primary factor affecting the piezoelectric readout of a tactile sensor. For the tactile sensor fabricated in Section 2, its sensitivity to the hardness of the material has been tested using the procedure depicted in Figure 7.

The test in Figure 7 conducts palpations on a set of elastic rubbers used for Shore A hardness calibration to evaluate the tactile sensor’s sensitivity to material hardness. With the hardness increasing from 30 HA to 88 HA, these rubbers are similar to human tissues in their mechanical hardness [22]. During the test in Figure 7, the vertical position of the sensor is optimized whenever the rubber is switched. The initial vertical position, Z_H_, is kept the same for the palpations on the same rubber. The palpations during Phase 2 are conducted five times at a single location for each piece of rubber. The pressing depth, D, is determined by the test in Section 3.2. D is kept the same throughout the whole test process in Figure 7. The average value of the five consecutive measurements is based on the tactile sensor’s readout voltage corresponding to the rubber’s hardness.

### 3.4. Capability to Detect Buried Rigid Objects

**Figure 8:**
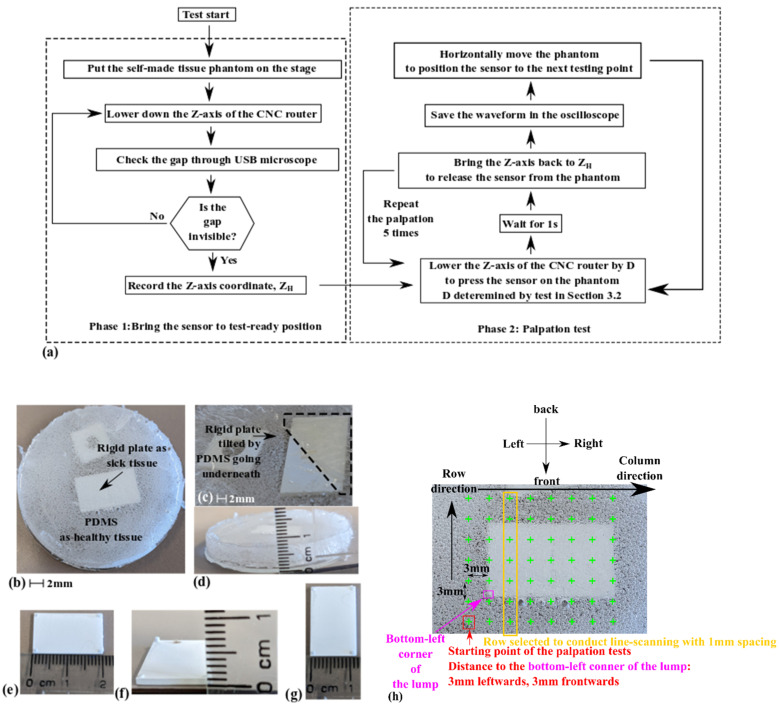
Method to test the piezoelectric tactile sensor effectiveness in palpation-based detection of buried rigid objects. (**a**) The flowchart of the method; (**b**) The top side of the self-made tissue phantom; (**c**) The bottom side of the phantom; (**d**) The thickness of the phantom; (**e**–**g**) The length, thickness, and width of the rigid plate as a hard tissue volume; (**h**) Map of the measurement locations.

According to existing research on similar topics [8,9,10,11,20,23,24,25,26,27,28,29,30,31], one promising application of tactile sensors for hardness differentiations during palpation is to detect the rigid abnormalities buried underneath normal tissues. These rigid volumes, such as lumps or tumors, usually have significantly higher hardness than the surrounding healthy tissues. The measurement test depicted in Figure 8 is dedicated to this purpose. 

The testing flow in Figure 8a is similar to the one in Figure 7. As shown in Figure 8b–g, a tissue phantom is prepared using Dow® Sylgard 184 PDMS kit. A 21 mm-by-12 mm-by-1 mm rigid plate is buried in 6 mm-thick PDMS to mimic an under-skin lump. The rigid plate is made by a 3D printing process of Formlabs^®^Rigid 10K resin. The PDMS is directly poured into a glass Petri dish and then cured. As shown in Figure 8c, when inspected from the backside of the tissue phantom, the bottom surface of the rigid plate has a varying color, indicating that the rigid plate is tilted after the PDMS is poured into the Petri dish. As shown in Figure 8d, the rigid plate has not extruded out of the PDMS top surface. 

The arrangement of the palpation tests is shown in Figure 8h. Two types of tests were conducted. The main test included measurements at 56 locations on the PDMS surface, covering the whole region with the rigid object buried underneath. The goal of the main test is to evaluate the tactile sensor’s capability to detect buried rigid lumps. The spacing between the neighboring testing locations was 3 mm: the same as the side length of the contact promoter shown in Figure 2a. The starting point of the palpation tests was 3 mm to the left and 3 mm in front from the surface position of the left-bottom corner of the hard plate underneath the PDMS. After the test at one location was completed, the tactile sensor was moved in a zig-zag manner to the next testing point, with a stepping interval of 3 mm. The palpation tests were conducted column-by-column, following the direction shown in Figure 8h. The second test was conducted to check if the tactile sensor could be used for hardness differentiation within an area smaller than its contact promoter. This test was only conducted in the third column, circled in the orange rectangle in Figure 8h. A step length of 1.1 mm was used, with 17 testing locations. In addition, the operations in Phase 1 of Figure 8a were conducted at the beginning of the second test.

The idea of using PDMS to imitate healthy tissues is inspired by the research of Boparai et al. [32]. The methodology for burying a rigid object into softer materials to evaluate the performance of a tactile sensor is inspired by the test conducted by Ju et al. [9]. They developed a piezoelectric tactile sensor using the frequency shift of electrical impedance peaks for hardness differentiation during the palpation processes of minimally invasive surgeries. The application potential of their tactile sensors was demonstrated by detecting the coin buried in a pig’s liver [9]. In their tests, palpations were conducted at the surface around the buried lump. Even though their experiment might have had reproducibility limitations because liver properties might vary from pig to pig, the methodology behind their setup has still been considered suitable for the proof-of-concept of tactile sensors [9].

## 4. Testing Results and Discussion

### 4.1. General Characteristics in the Voltage Readout for Palpation

**Figure 9:**
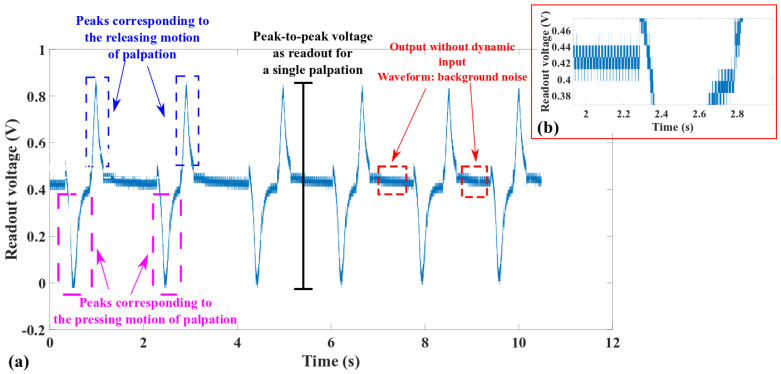
Representative readout during the palpation process of polymeric piezoelectric tactile sensor. (**a**) The waveform of six consecutive palpation operations. (**b**) Zoom-in view of the background noise.

Figure 9 presents a representative readout waveform implemented by the setup in Section 3.1 to discuss some general characteristics observed in the testing results.

Similar to the readout of other piezoelectric tactile sensors [33], the waveform in Figure 9a reflects the strengths and weaknesses of piezoelectric sensing. The piezoelectric effect is not suitable for static measurements. Instead, it works well in sensing the time-domain dynamics of an input excitation. This is why the output voltage in Figure 9a shows only background noise when the tactile sensor is above the target surface or pressed statically against the target surface. Similarly, peaks with a 180-degree phase difference appear in pairs during the motions to press or lift the piezoelectric tactile sensor. These peaks disappear when the motions of pressing or releasing end. In Figure 9a, the DC offset is around 0.9 V, indicating that the tactile sensor has already been pressed against the target surface before the palpation cycles leading to the voltage peaks in Figure 9a are conducted. To minimize the impact of such experimental conditions on the evaluation of the tactile sensor’s performance, this paper considers the voltage difference between the peak corresponding to the pressing and the peak corresponding to releasing as the readout for a single palpation. Figure 9b shows a zoom-in view of the static background noise with a peak-to-peak amplitude of around 40 mV. In addition to the intrinsic electrical noise of the readout electronics, other sources of noise are due to the DC power supply and the non-shielded interconnected wires. 

### 4.2. Sensitivity to Pressing Depth during Palpation

**Figure 10:**
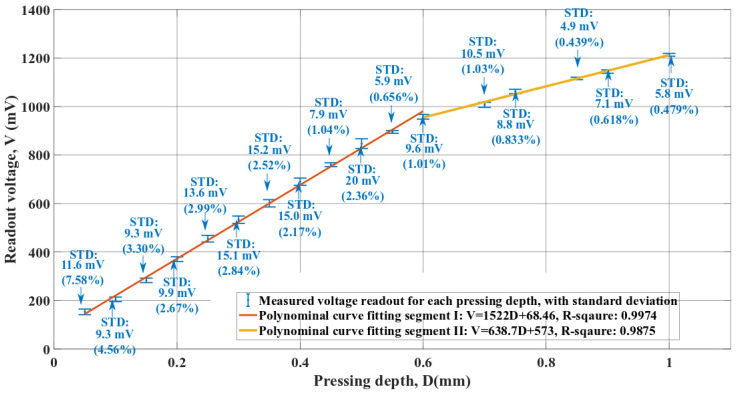
The piezoelectric tactile sensor readout output for input displacement from 50 µm to 1000 µm, with an increment step size of 50 µm.

Results of the test to evaluate the tactile sensor’s sensitivity to pressing depth during palpation are shown in Figure 10.

In Figure 10, the maximum standard deviation of the measured voltage readout is around 7.5%. The corresponding pressing depth is 50 µm. For other pressing depth values, the standard deviations are below 5%. Based on the testing flow shown in Figure 6 of Section 3.2, the minor standard deviation in Figure 10 indicates that the tactile sensor has a relatively consistent response to the same conditions during repeated operations. In addition, the lower and upper limits of the neighboring voltage readouts do not overlap in Figure 10. This observed result demonstrates that the fabricated tactile sensor can detect a pressing depth difference of around 50 µm.

In Figure 10, the measured voltage readouts can be divided into two linear regimes, separated by a transition pressing depth value of 600 µm. The two regions are linear, with good correlation coefficients but different sensitivities. Region I (small deformations) has a higher slope than Region II, indicating that the tactile sensor has an input-dependent sensitivity to the pressing depth during palpation. Similar characteristics have been reported for other tactile sensors using elastic polymers as structural materials [34]. The reduced slope of segment II could indicate that the tactile sensor is approaching its elastic deformation limit. Stainless steel has significantly higher stiffness than the structural material of the tactile sensor and the polymeric materials used in the other two tests. The characteristics in Figure 10 can be considered as mainly related to the structural design and material properties of the tactile sensor. The voltage readouts in Figure 10 indicate that the tactile sensor faces an increased risk of irreversible deformation and damage when the pressing depth is larger than 600 µm. As mentioned in Section 3.2, the characterization of the tactile sensor’s response to the pressing depth allows the authors to determine a proper pressing depth value used by the other two tests to protect the tactile sensor. Therefore, a pressing depth value of 500 µm is selected for the other two tests, with a 100 µm ‘safe distance’ from the knee-point in Figure 10.

The measurement results in Figure 10 indicate a non-zero voltage output when the pressing depth value is zero: very close to the background noise amplitude of 40 mV in Figure 9. In addition, this non-zero voltage readout for the zero pressing depth could be the result of the tactile sensor being slightly pressed on the stainless steel stage during Phase 1 in Figure 6. The corresponding influence on evaluating the tactile sensor sensitivity to the pressing depth is limited. As already discussed in this section, any sensitivity to the pressing depth derived from Figure 10 will primarily represent the tactile sensor’s performance when related to the structural design and material properties. Such sensitivity is independent of the hardness of the palpation target. Based on the slopes of Segment I and Segment II in Figure 10, the tactile sensor has a sensitivity of 1522 mV/mm for small pressing depth values and a sensitivity of 638.7 mV/mm for larger ones. These two derived sensitivity values are compared with the sensitivity of tactile sensors for similar applications. Mei et al. developed a polymeric sensor with a sensitivity of 10 mV/mm using silicone as the structural material [35]. The flexible tactile sensor developed by Chen et al. has a sensitivity of 64.1 mV/mm [36]. The tactile sensor developed by Khodambashi et al. has a sensitivity of 14.5 mV/mm. Recently, Zhang et al. developed a tactile sensor for similar applications with a sensitivity of 1010 mV/mm [37]. The piezoelectric tactile sensor fabricated by Zhou et al. has a sensitivity of 730 mV/mm [38]. The performance of the tactile sensor presented in this paper can be considered comparable with existing devices for similar applications.

### 4.3. Sensitivity to the Hardness of Palpation Target

**Figure 11:**
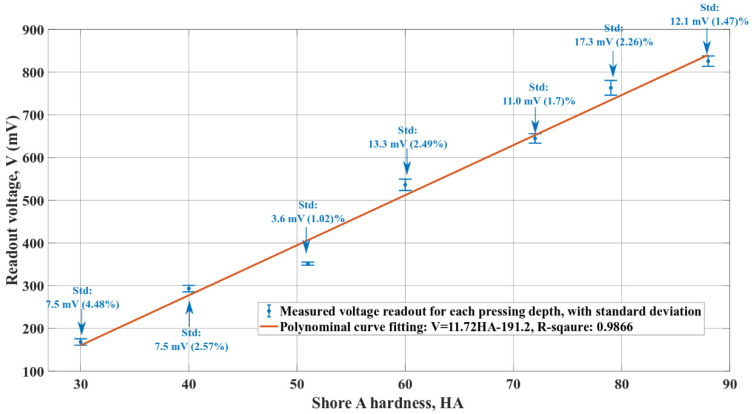
The tactile sensor sensitivity to various hardness levels of the palpation targets.

The result of the tests that evaluated the tactile sensor sensitivity to the hardness of the palpation target is shown in Figure 11. All palpation tests to obtain Figure 11 have used a pressing depth value of 500 µm.

The experimental standard deviations that were recorded for the measurement results in Figure 11 are below 5%. Based on the testing procedure in Figure 7 of Section 3.3, the minor standard deviations in Figure 11 show that the tactile sensor has a relatively consistent response to the same test conditions during repeated operations. These standard deviations also demonstrate that the measurement variations are smaller than the differences between the two neighboring measurements for different hardness levels. Hence, the measurements in Figure 11 indicate that the fabricated tactile sensor can detect a hardness difference of around 8.2 HA for soft materials.

The seven voltage readouts in Figure 11 are within a similar range to the twelve voltage readouts of the pressing values, which are smaller than 600 µm in Figure 10. Table 3 compares the voltage readouts in Figure 11 with their closest counterparts in Figure 10. 

As shown in Table 3, though the Shore A hardness calibration rubbers are significantly softer than the stainless-steel stage, a voltage readout with similar values can still be achieved by increasing the pressing depth during the palpation. Such a phenomenon reflects some characteristics that have also been mentioned in other research about piezoelectric tactile sensors for hardness differentiation [10,23]: the output voltage of a piezoelectric tactile sensor during a palpation operation depends on both the pressing depth value and the difference in material hardness between the target object and the tactile sensor. The same voltage output signal can be obtained by pressing deeper onto a softer target or pressing for less distance into a harder target. Softer materials require larger increments in the pressing depth. 

In Figure 11, the voltage readout dependence on the hardness scale can be fitted on a first-order polynomial with a good correlation coefficient. The slope of the fitted curve, 11.72 mV/HA, represents the tactile sensor’s sensitivity to the hardness of the palpation target for a fixed palpation depth of 500 µm. The fitted curve in Figure 11 has a non-zero voltage output when the material hardness is zero, which could be related to the vertical re-positioning of the tactile sensor during Phase 1 in Figure 7. 

### 4.4. Capability to Detect the Lump Buried in Soft Material

**Figure 12:**
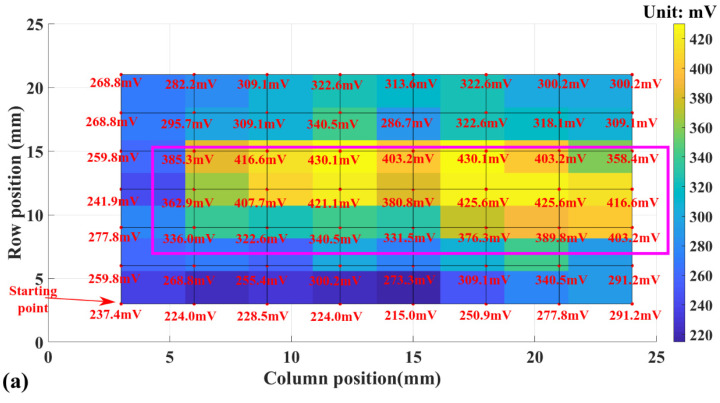

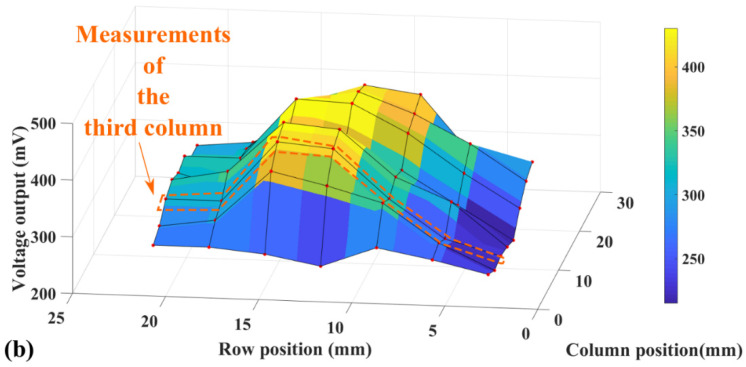
Voltage readout mapping to the area scanning of palpations on a self-made tissue phantom. (**a**) 2D view of the measurement result, (**b**) 3D view of the measurement result.

The voltage readout of the primary palpation tests to detect the lump buried in PDMS is shown in Figure 12. All palpation tests have used a pressing depth of 500 µm.

The red dots in Figure 12 correspond to the grid points represented by the green crosses in Figure 8h. The voltage readout for each red dot is labeled in Figure 12a. Since all the readouts are within the range in Figure 11, it becomes suitable to analyze them using the fitted curve in Figure 11. In Figure 12a, the output voltage values at the PDMS surface locations above the buried rigid plate in Figure 8h are circled out by the pink rectangle. The readouts within the pink rectangle have an average value of 388.91 mV, with a standard deviation of 34.85 mV. This average value corresponds to a Shore A hardness of 49.49 HA. For the voltage readouts on the PDMS surface with no rigid lump buried underneath, their average value is 282.76 mV, with a standard deviation of 34.02 mV. This average value corresponds to a Shore A hardness of 40.44 HA. The average difference in the detected material hardness within and outside the pink rectangle is around 9 HA. 

Existing studies of the cured Sylgard 184 PDMS elastomer show that the hardness of the material is dependent on the processing conditions. Johnston et al. systematically studied the impact of material processing conditions on the mechanical properties of the Sylgard 184 PDMS elastomer, reporting hardness values ranging from 44 HA to 54 HA [39]. The hardness of the cured Sylgard 184 PDMS detected by the tactile sensor is slightly below the lower limit reported by Johnston et al. One possible reason for this is that the PDMS had not been degassed, as shown in Figure 8. The looser control over the processing conditions may generate variabilities at the hardness of the cured PDMS. Nevertheless, the measurement results in Figure 12a indicate that, even with increased variability in the PDMS hardness, the rigid plate underneath could still be detected by the tactile sensor during palpations on the PDMS surface. The voltage output of the tactile sensor has been increased due to the localized increment in material hardness caused by the buried rigid lump.

Figure 12b presents more detail about the increment in material hardness caused by the buried rigid plate and the corresponding effect on the voltage readouts of the tactile sensor. It can be straightforwardly observed that, for testing locations on the PDMS surface above the buried rigid lump, locations closer to the (0,0) point in Figure 12b have smaller voltage readouts, indicating a lower equivalent material hardness. Such a non-uniform increment in material hardness could be related to the tilting of the buried rigid plate, as shown in Figure 8c. The part of the rigid plate closer to the starting point in Figure 8h is buried deeper in the PDMS, reducing the increment in the equivalent material hardness and the corresponding voltage readout values. In addition, in Figure 12b, the testing points on PDMS closer to the upwardly tilted side of the rigid plates have higher voltage readouts than the testing points closer to the downwardly tilted side. The difference indicates that the equivalent hardness of the surrounding PDMS is also affected by the rigid plate and its final orientation.

**Figure 13:**
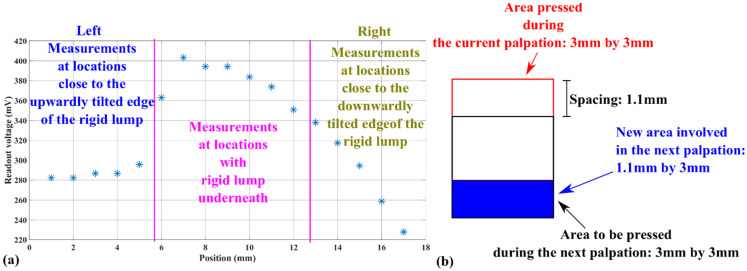
Voltage readout corresponding to the line scanning of palpations on a self-made tissue phantom. (**a**) The voltage readout, (**b**) The schematic depicting how the tactile sensor is moved between each palpation.

The result of the second series of palpation tests conducted only along the third column in Figure 8h is shown in Figure 13.

In Figure 13a, the voltage readouts follow a trend similar to the one for the third column in Figure 12b (circled in orange dashed lines). The difference between the voltage readouts in Figure 12b and Figure 13a could be related to the limited accuracy in the vertical re-positioning of the tactile sensor. In Figure 13a, the non-uniform influence of the tilted rigid plate buried in the PDMS on the equivalent hardness and the voltage outputs is still observable. As shown in the left part of Figure 13a, the upwardly tilted side is closer to the PDMS surface, leading to a more significant increment to the equivalent hardness. Hence, this upwardly tilted side can be detected more sharply from the voltage readouts. On the contrary, the increment in the equivalent hardness related to the downwardly tilted side is more limited. Consequently, the voltage readout corresponding to the border on this side has a smaller difference from the voltage readout corresponding to the surrounding PDMS, as shown in the right part of Figure 13a.

In addition, as mentioned in Section 3.4, the second series of palpation along the third column used 17 testing points with a spacing between neighboring points of around 1.1 mm. As shown in Figure 13b, due to this setup, only a 1.1 mm-by-3 mm new area was involved in each palpation test. Hence, the gradually decreasing voltage readouts in the right part of Figure 13a suggest that the tactile sensor can detect hardness differences within an area smaller than the area of its contact promoter.

## 5. Conclusions

This paper presents the design, fabrication, and experimental testing of a polymeric piezoelectric tactile sensor, with dimensions commensurable with existing catheter diameters. The fabrication process is simple and reliable, combining the 3D printing of elastic resin and laser micromachining of a piezoelectric polymeric thin film. Multiple experimental characterizations have been conducted for the tactile sensor to evaluate its sensitivity to the pressing depth, characterize the palpation response to different hardness levels of the target, and detect and identify the borders of a rigid object buried underneath a softer layer.

The tactile sensor has exhibited a sensitivity of 1520 mV/mm for pressing depth values smaller than 600 µm and a sensitivity of 643 mV/mm for pressing depth values between 600 µm and 1000 µm. A sensitivity of 11.72 mV/HA has also been observed for calibrated soft materials, with hardness levels similar to biological tissues. Palpation tests at grid-point locations on a self-made tissue phantom have validated the tactile sensor’s capability to detect buried lumps through the difference in hardness response measured on the surface. During the same series of tests, it was also found that the tactile sensor could detect hardness differences within an area smaller than the size of its contact promoter. The performance of the tactile sensor during the tests can be considered comparable with other existing tactile sensors. At the same time, its simple, low-cost fabrication process makes it a good candidate for palpation-based tissue techniques for various medical applications.

The proof-of-concept in this paper can serve as the basis for two types of future work. The first direction is towards scaling down the dimensions of the tactile sensor to facilitate integration with medical robotic systems for minimally invasive surgery or catheter-based diagnosis. The second direction is towards the design of tactile sensor arrays integrated with electronics in wearable systems for augmented sensing systems during medical operations.

## Figures and Tables

**Table 1:** Material, equipment, and processes used to fabricate the piezoelectric tactile sensor.

Component	Material	Material Vendor	Processing Method	Equipment	Service Provider
Top elastic layer	Flexible 80	Formlabs, USA	Stereolithography 3D printing	Formlabs^®^3L 3D printer	3D Shop Canada
Piezoelectric layer	PVDF-TrFE thin film	Poly-K, USA	Laser micromachining	Oxford^®^laser micromachining system	The authors’ lab
Electrodes	Silver-based conductive ink	Circuit Scribe, USA	Shadow-mask-based screen printing	Manual operation	The authors’ lab
Shadow mask	Copper-polyimide composite	Dupont, USA	Shadow-mask-based screen printing	Oxford^®^laser micromachining system	The author’s lab
Bottom elastic layer	PDMS thin film	HNXCK, China	Laser micromachining	Oxford^®^laser micromachining system	The authors’ lab
Device base	Rigid 4K	Formlabs, USA	Stereolithography 3D printing	Formlabs^®^3L 3D printer	3D Shop Canada

**Table 2:** List of hardware equipment used to test the polymeric piezoelectric tactile sensor.

Equipment	Module
CNC router	Genmistu^®^3018-Pro
Charge amplifier readout circuit	Analog Device^®^CN0350 piezoelectric sensor evaluation board
Oscilloscope	Siglent^®^SDS1200X-E oscilloscope (Bandwidth: 200 MHz, Sampling rate: 1 kSa/s)
USB Microscope	Bysameyee^®^HD 2K 2MP USB Microscope, 40X to 1000X Magnification Digital Microscope Camera Inspection Endoscope
DC power supply	Protek^®^PL-3003S DC regulated power supply

**Table 3:** Comparison of the readout voltage values in Figure 10 and Figure 11 with the minimum difference.

Palpation Tests Using a Pressing Depth of 500 µm on Rubbers with Different Hardness (Figure 11)		Palpation Tests on a Stainless Steel Stage with Different Pressing Depth Values (Figure 10)	
Rubber Hardness (HA)	Readout Voltage (mV)	Palpation Depth Values (µm)	Readout Voltage (mV)
30	168.2	50	153.2
40	293.2	150	283.3
51	351.6	200	307.7
60	536.2	300	533.4
72	644.8	350	600.9
79	763.2	450	760.2
88	825.7	500	847.1

## Data Availability

The measurement results are available upon request.
